# Practical management of nocturia in urology

**DOI:** 10.4103/0970-1591.42607

**Published:** 2008

**Authors:** Myeong Heon Jin, Du Geon Moon

**Affiliations:** Department of Urology, Korea University College of Medicine, Seoul, Korea

**Keywords:** Nocturia, review, urology

## Abstract

Until last decades, nocturia has been considered as an irritative symptom of benign prostatic hyperplasia (BPH), but the nocturia is unresponsive symptoms to various modalities of BPH treatment. More recently, it has been recognized that the prostate is not so quite important as previously believed, as nocturia is equally common in women. The understanding of nocturia has been much changed in last decade; it is a highly prevalent condition, and symptoms in men and women are really no different either quantitatively or qualitatively. The successful introduction of desmopressin (l-deamino-8-D-arginine vasopressin, DDAVP) to decrease nocturnal urine output in severe nocturia which resistant to conventional BPH treatment and in women demonstrated new perspectives in management of nocturia. We reviewed the definition and etiologies of nocturia, offering the current diagnostic procedures and standards of care.

## DEFINITION

Recently, nocturia has only been recognized as a separate clinical entity within the lower urinary tract symptom complex.[1] The pathophysiology of nocturia is multifactorial and can be complex and its cause remains unclear in a significant number of patients.[2] The growing recognition that nocturia is a condition in its own right was reflected in the meeting ‘Nocturia - towards a consensus’ that was convened in Athens in 1998.[1] The International Continence Society (ICS) defines nocturia as “the complaint that the individual has to wake at night one or more times to void.”[1] The ICS and also defines nocturnal urine volume (NUV) as “the total volume of urine passed between the time the individual goes to bed with the intention of sleeping and the time of waking with the intention of rising.” Thus, NUV does not include the last void before going to bed, but it does include the first morning void. An important consideration of nocturia is whether the patient is awakened by the need to void, or if the patient voids after being awakened for some other reason. Every patient of nocturia does not need treatment. Patients who are awakened by nocturia and suffered from lack of sleep are candidates for evaluation. In clinical practice, most people do not seek treatment for 1-2 times of nocturia. So, severe nocturia >2-3 times per night will be evaluated. Sleep time varies by individual, but on average, it is 8 h per night. This can affect the evaluation of nocturia, because the number of nocturnal voids depends partly on how many hours an individual actually sleeps. Shift workers may have a variation in their nighttime, and the same definition exists for them. This is important, because more than 15 million of the general populations are shift workers who sleep during the day.[3]

## PREVALENCE

**Figure 1:**
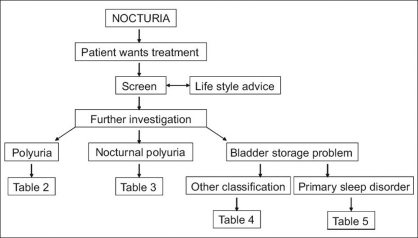
Prevalence rate of nocturia of ≥2 times per night in different age decades[8]

Early prevalence studies used varied definitions of what might constitute a problem, whereas later studies have conformed to the ICS definition and related nocturnal frequency to its associated impact. Although there are limited data on the frequency of voiding and volume voided nocturnally across all age groups,[4] prevalence in community-dwelling populations has been measured and is typically present in >50% of both men and women aged >60 years. The prevalence increases with age such that the vast majority of any population of men and women of 80 years and over will rise at least once at night to empty their bladder.[5–7] The descriptive epidemiological data on nocturia indicate that this symptom increases with age and the steepest increase is in older groups (>65 years). There were no large differences in the prevalence of nocturia between men and women; however, there is a tendency for young women to have this symptom more often than young men, and for very old men to have it more often than very old women.[8] Typical ranges for ≥2 nocturia episodes/night are 5-15% for those aged 20-50 years, 20-30% for those aged 50-70 years, and 10-50% for those aged ≥70 years [Figure 1].[8]

## IMPACT OF NOCTURIA

Nocturia is relatively common among the elderly and becomes increasingly prevalent with age, with profound influences on health and quality of life (QoL). Sleep fragmentation and disruption may result in daytime sleepiness, tiredness, mood changes, and cognitive dysfunction with poor concentration and performance.[9,10] More than 60% of men and women reported that nocturia had a negative effect on their QoL.[8] In fact, nocturia is often quoted as the most bothersome of lower urinary tract symptoms, and there is an association between the extent of impact and the frequency of nocturnal voiding.[11–14] A quarter of community-dwelling individuals aged 65-74 years and a third of those of aged ≥75 years experience one or more falls every year.[15] A quarter of the falls occur at night, and more than a quarter of these nocturnal falls occur in relation to toilet visits.[16]

Recently, the impact of nocturia on productivity at work, vitality and QoL were assessed among professionally active individuals in Sweden.[17] Over 200 individuals reporting, on average, more than two voids per night were compared with a group of age- and gender-matched controls without nocturia. This study clearly showed that lack of sleep caused by excessive nighttime voiding leads to lower energy levels (vitality), impaired work-related productivity, and reduced QoL compared with controls. Nocturia also has been known to be linked to a heightened risk for traffic accidents, morbidity, mortality, and significant health costs to both the patient and the physician.[18] Until recently, there was no condition-specific quality-of-life instrument which assessed the impact of nocturia upon well-being. The recently developed nocturia quality-of-life questionnaire measurement tool consists of 13 items assessing productivity, level of activity impairment, energy, fatigue, and worry. The score is able to discriminate between men experiencing one, two, or three or more episodes of nocturia. It has not yet been validated for use in women.[19]

## DIFFERENTIAL DIAGNOSIS

Because the ICS definition of nocturia does not suggest causation, it is necessary to consider the reasons of nocturia in each individual. The underlying pathophysiologic process of nocturia comprises four main conditions: (i) global polyuria, (ii) nocturnal polyuria, or nocturnal urine overproduction, (iii) bladder storage problems, or decreased nocturnal bladder capacity (NBC), and (iv) mixed.

### Global polyuria

Polyuria is continuous overproduction of urine not limited to sleep hours and defined as 24-h urine output of more than 40 ml/kg. Polyuria occurs in response to the increased fluid intake, which in turn leads to excessive urine production. As a result, polyuria is usually associated with increased urinary frequency during both daytime and nighttime. The common causes of global polyuria are diabetes mellitus, diabetes insipidus (DI), and primary thirst disorders. The less common form of diabetes, DI, is a disorder of water balance in the body. Inappropriate excretion of urine may lead to polydipsia to prevent circulatory collapse. Central DI is caused by deficient synthesis of antidiuretic hormone secondary to the loss of neurosecretory neurons in the hypothalamus or posterior hypophysis. In nephrogenic DI, the pituitary gland produces a normal amount of antidiuretic hormone, but the kidneys do not respond appropriately to it.[20] Diagnosis is made by overnight water deprivation to determine whether the urine becomes more concentrated. If the first morning void is not highly concentrated, DI is diagnosed. Central DI usually can be treated with desmopressin.[21] Nephrogenic DI may be treated with judicious regulation of fluid intake. If the water deprivation test is normal in a patient experiencing polyuria, the diagnosis is a primary thirst disorder such as polydipsia, which may be either dipsogenic or psychogenic. In polydipsia, the patient demonstrates normal renal concentrating capacity. Although not classified, polyuria could be observed in patients taking excessive fluid to maintain general health from medical misknowledge.

### Nocturnal polyuria or nocturnal urine overproduction

Nocturnal polyuria is defined as an increase in nighttime urine production with a corresponding decrease in daytime urine production, resulting in a normal 24-h urine volume. Similar to the pathophysiology of nocturnal enuresis, a disruption in the diurnal secretion patterns of arginine vasopressin (AVP) has been proposed for nocturia.[20] In 2004, the authors studied the circadian variation of AVP and urine output in patients with severe nocturia.[22] Compared with the normal control, the nocturia patients had no diurnal variation in urine output and greater nocturnal urine production, associated with a lack of nocturnal increase in AVP level. Secondary causes of nocturnal polyuria include congestive heart failure; diabetes mellitus; peripheral edema due to venous stasis or lymphostasis, nephritic syndrome, hepatic failure, or hypoalbuminemia; or lifestyle patterns such as excessive nighttime drinking. In clinics, patients of nocturnal polyuria caused by excessive nighttime drinking are very common due to the nighttime burden of pills for multiple combined disease or risk factors of systemic disease. The increased airway resistance that is associated with obstructive sleep apnea may also lead to nocturnal polyuria because of increases in renal sodium and water excretion that are mediated by elevated plasma atrial natriuretic peptide levels.[20] Diuresis via the inhibition of AVP can be caused by prostaglandin E-2, atrial natriuretic peptide, hypercalcemia, hypokalemia, lithium, and tetracycline. Patients being treated with lithium or tetracycline may present with what appears to be nephrogenic DI; therefore, a thorough medical history is necessary for differential diagnosis. The formula for calculating nocturnal polyuria index (NPi) is simply NUV divided by 24-h urine volume [Table 1]. If 24-h urine production is within normal limits, NPi greater than 35% is indicative of nocturnal polyuria.[23]

**Table 1:** Formulas for nocturia evaluation

Formula	Analysis
*Nocturia index*	
Ni = NUV/MW	Ni > 1: novturia is due to NUV exceeding MVV
*Nocturnal polyuria index*	
NPi = NUV/24 hV	NPi > 35%: diagnosis is nocturnal polyuria
*Nocturnal bladder capacity index*	
Ni - 1 = PNV^a^	NBCi > 0: nocturia occuring at volumes < MVV
NBCi = ANV - PNV	

a Round up to next higher number if Ni - 1 is not an integer

### Bladder storage problems or decreased nocturnal bladder capacity

Patients with nocturia who do not have either polyuria or nocturnal polyuria according to the above criteria, will most likely have a reduced voided volume or a sleep disorder. NBC is defined as the largest voided volume during the hours of sleep. Decreased NBC can be related to decreased maximum voided volume (MVV) or decreased NBC.[24] Decreased NBC can be related to prostatic obstruction, nocturnal detrusor overactivity, neurogenic bladder, primary bladder pathology such as cancer (bladder, prostate, or urethra), learned voiding dysfunction, anxiety disorders, certain pharmacological agents, or bladder and ureteral calculi.[24] Some mathematical indices have been suggested to describe this situation.

NBC index (NBCi) is a more complicated formula that addresses voids at night in patients with decreased NBC. NBCi is actual number of nightly voids (ANV) minus predicted number of nightly voids (PNV) [Table 1]. PNV is derived by calculating nocturia index (Ni) (NUV/MVV) and subtracting 1. NBCi greater than 0 indicates nocturia occurring at volumes less than MVV.

### Mixed nocturia

A significant number of nocturia cases are related to more than 1 etiology. Mixed nocturia is a combination of nocturnal polyuria and decreased NBC, and it is more common than many realize. In a study of 194 patients in whom nocturia was a chief or significant complaint, 7% were determined to have simple nocturnal polyuria, 57% had decreased NBC, and 36% had a mixed etiology, combining the first two etiologies.[25] Thus, nocturnal polyuria was a factor in nocturia in 43% of the patients evaluated. It was concluded that the etiology of nocturia was multifactorial and often unrelated to an underlying urological condition, such as prostatism. Mixed nocturia is diagnosed through the maintenance and analysis of bladder diaries. Through the formulas noted in Table 1, it is possible to assess the relative contribution of nocturnal polyuria and decreased NBC.

## CLINICAL ASSESSMENT OF NOCTURIA

As with any patient, a detailed history of the problem is required. It is essential to establish what is normal for the patient and whether there has been a change from normal. If there has been a change, either sudden or gradual then the amount by which the patient is bothered by the symptom should dictate whether further investigation of the problem is required. As in the management of all lower urinary tract symptoms, there should also be an assessment of the patient's own desired objectives following treatment. This will allow a problem-based approach to management. An initial screen should include a detailed history, including questions relevant to voiding behavior, medical and neurological abnormalities, and sleep disturbance, as well as information on relevant surgery or previous urinary infections. A simple urine test, e.g. dipstick or urine analysis, should be undertaken to exclude any relevant pathology. The patient should also have a physical examination. During physical examination, patients should be screened for edema, age-related changes of the genitalia, and prolapse. Neurologic examination should include evaluation of anal sphincter tone, the bulbocavernosus reflex, and sensation in the sacral dermatomes [Table 2][1].

**Table 2:** Causes of polyuria

*Diabetes mellitus*
Insulin dependent (Type I)
Insulin independent (Type II)
*Diabetes insipidus*
Pituitary
Renal
Gestational
Primary polydipsia (psychogenic, dipsogenic, or iatrogenic)

**Figure 2:**
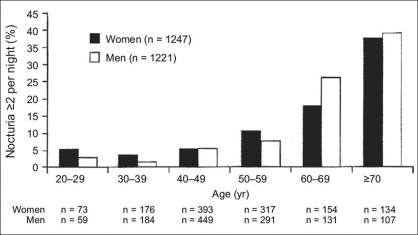
Algorithm for investigating nocturia

The assessment of nocturia can be described by a simple algorithm [Figure 2]. This considers the possibility that the patient may present to the physician specifically because of nocturia, or may present with another condition, while also having nocturia. The principal diagnostic tool for use in the assessment of nocturia is the voiding bladder diary. Based on information about urinary frequency and volume that is recorded by the patient in the diary, a physician can classify the patient as having polyuria, nocturnal polyuria, or bladder storage problems. Information that should be collected and recorded in the voiding bladder diary includes timing of voids, number of voids, and volume of urine voided. Fluid intake volume and time of fluid intake should also be recorded. When collecting these data, patients include the first morning void in the NUV because the kidneys excreted this amount during sleep. However, the first morning void is considered a normal episode and is not included with the number of nightly voids.

## TREATMENT

### Lifestyle factors

Managing nocturia is best based on an approach that targets the underlying causes, but changes in several empirical lifestyle factors might be effective. Restriction of caffeine and alcohol intake may help some patients with nocturia.[1] Fluid restriction before bedtime may help to alleviate the disorder, especially in patients who also have urgency incontinence.[26,27] Griffiths *et al.*,[27] studied 128 incontinent elderly patients and found that evening fluid restriction reduced nocturnal urine excretion by a small amount in those with severe urgency incontinence. Some data suggest that this method alone may not be ideal for alleviating nocturia in the elderly. For instance, Asplund and Åberg[28] found that nearly half of those who woke ≥3 times a night to urinate already restricted their fluid intake in the evening. In cases of nocturnal polyuria, the urine output cannot be reduced by restricting fluid intake, because of a lack of circulating AVP and an inability to respond with inhibition of the diuresis after fluid restriction. Also fluid restriction before bedtime is rarely effective if nocturia is the result of gravity-induced third spacing of fluid in the lower extremities. In this situation, fluid is mobilized when the patient assumes a recumbent position. Compression stockings, if worn by the patient during ambulatory periods, may prevent fluid accumulation in the legs, thus helping to diminish nocturnal urinary output. Diuretics given during the late afternoon or early evening may also help to decrease third spacing of fluid. If given later during the evening, however, diuretics may increase nocturia [Table 3].[1,29]

**Table 3:** Causes of nocturnal polyuria

*Water diuresis*
Circadian defect in secretion or action of antiduretic hormone
Primary (idiopathic)
Secondary (excessive evening intake of fluid, caffeine, alcohol)
*Solute/water diuresis*
Congestive heart failure
Autonomic dysfunction
Sleep apnoea syndrome
Renal insufficiency
Estrogen deficiency

### Surgery

Transurethral prostatectomy/transurethral incision of the prostate may be appropriate when obstruction related to the prostate is believed to be a significant causative factor of nocturia. Surgical correction of pelvic organ prolapse, sacral nerve neuromodulation, detrusor myectomy, and clam cystoplasty are options for the treatment of patients with intractable nocturia associated with overactive bladder (OAB).

### Pharmacotherapy

To date, desmopressin, a synthetic analog of AVP, is the only available antidiuretic drug, and has been used for over 30 years in the treatment of disorders such as DI and primary nocturnal enuresis (PNE). As it is now recognized that nocturnal polyuria is also a major component of nocturia, the place of desmopressin for treating nocturia is gaining acceptance. Several clinical trials investigating the benefits of desmopressin therapy for patients with nocturia have been completed.[30–33] These randomized, double-blind, placebo controlled, NOCTUPUS trials assessed the short- and long-term efficacy, and safety of desmopressin in nocturia in men[30] and in women[31] treated with oral desmopressin at bedtime. In the short-term (3 weeks) trials, 33% of males and 46% of females desmopressin-treated patients showed a ≥50% reduction in the mean number of voids per night relative to baseline (*P* < 0.001 and *P* < 0.0001, respectively, compared with placebo). All other efficacy endpoints, including the mean number of nocturnal voids and the duration of the first sleep period until the first nocturnal void, showed a highly significant difference in favor of desmopressin compared with placebo.[30–32] Quality of life analyses also indicated a greater improvement in terms of prevalence, bother, and problems caused by nocturia, in patients treated with desmopressin compared with placebo. Of patients completing the short-term NOCTUPUS studies, 88% entered the 10-12-month extension study.[33] At 12 months, the proportion of patients with a ≥50% reduction in the number of nocturnal voids compared with baseline increased from 37 to 67% in males, and from 46 to 67% in females. Overall, efficacy in all endpoints and the improvement in QoL were maintained during long-term treatment. The main adverse effect of treatment with this drug is a dilutional hyponatremia, the incidence of which was found to be 7.6% (95% CI 3.7-15.1) in a recent metaanalysis.[34] Treatment of nocturia in elderly patients with desmopressin should only be undertaken together with careful monitoring of the serum sodium concentration. A significant fall in serum sodium concentration can occur within 72 h, and the risk increases with increasing age (over 65-year group at most risk) and decreasing baseline serum sodium concentration.[35] In patients of risk factors for hyponatremia, the sodium concentration should be checked within 1 week of trial and the dose should be titrated. Patients with a baseline serum sodium concentration below normal range should not be treated. In long-term treatment of nocturia, desmopressin administration for 1 year in elderly patients did not affect baseline AVP secretion, and gradually decreased serum sodium and it might induce hyponatremia even in patients without initial hyponatremia. For long-term desmopressin administration serum sodium should be assessed carefully, at least at 6 months [Tables 4 and 5].[1,36]

**Table 4:** Causes of problems related to bladder storage

*Reduced functional bladder capacity (e.g. significant postvoid residual)*
*Reduced nocturnal bladder capacity*
*Detrusor overactivity*
Neurogenic (e.g. multiple sclerosis)
Nonneurogenic
*Bladder hypersensitivity*
Bladder outlet obstruction with postvoid residual urine
Urogenital ageing

**Table 5:** Sleep disorders potentially related to nocturia

Insommia
Obstructive and central apneoa syndrome
Periodic legs syndrome
Restless legs syndrome
Parasomnias
Sleep disorders related to medical diseases, e.g. chronic obstructive
lung disease, cardiac disease, etc.
Sleep disorders related to neurological diseases, e.g. Alzheiemer's
Parkinson's and nocturnal epileptic seizures

Patients with nocturia associated with detrusor overactivity, a component of overactive bladder syndrome and the common symptom of urgency incontinence,[37] may respond to drugs that affect bladder contractility. These drugs include the antimuscarinic agents oxybutynin and tolterodine, which can substantially reduce the voiding frequency in patients with overactive bladder.[38,39] Solifenacin and other new antimuscarinic drugs in development for treating overactive bladder, e.g. darifenacin, have potentially better safety and tolerability profiles than the older antimuscarinic agents.[40,41]
